# Surface damage from perpendicular and oblique bullet impacts in stone

**DOI:** 10.1098/rsos.220029

**Published:** 2022-07-06

**Authors:** Oliver Campbell, Tom Blenkinsop, Oscar Gilbert, Lisa Mol

**Affiliations:** ^1^ School of Earth and Ocean Sciences, Cardiff University, Cardiff, UK; ^2^ Department of Geography and Environmental Management, University of the West of England, Bristol, UK

**Keywords:** photogrammetry, bullet impact, crater morphology, oblique impact, asymmetry, heritage

## Abstract

Controlled experiments were conducted to investigate the surface damage caused by perpendicular and oblique impacts of bullets into sandstone and limestone targets. Individual bullets fired in conditions simulating modern rifles at typical combat distances excavated craters with diameters from 22 to 74 mm and depths from 3 to 24 mm. Limestone target craters were up to twice as large and deep as those in sandstone. These craters have a complex shape consisting of a central excavation surrounded by a shallow dish, compared to the simple bowl shape of most sandstone impacts. Radial fractures extending to the edge of the target block were common in limestone targets. Impacts at an angle of 45° to the surface in both rock types result in asymmetric craters. Two common types of intermediate cartridge (ammunition) were compared: the steel-tipped 5.56 × 45 mm NATO projectile generally produced larger and deeper craters than the 7.62 × 39 mm projectile that is commonly fired from AK-47 rifles, despite having approximately half the mass of the latter. These results characterize the sort of damage that can be expected at many sites of cultural significance involved in contemporary conflict zones, and have important implications for their conservation: for example building stone with low tensile strength is likely to sustain more damage and be at risk of greater deterioration.

## 1. Introduction

As contemporary armed conflicts shift towards more urbanized areas, the risk of damage to non-military targets, such as homes, shops and places of worship, increases. The most dramatic manifestations of armed conflict are destruction from explosives, rockets and heavy artillery, particularly to sites of cultural significance, e.g. the ideologically driven destruction of Mosul and Palmyra by Islamic State (IS)/Da'esh [1]. By contrast, bullet impacts and shrapnel damage are less obvious, and commonly overlooked in damage assessment. There are few studies on the quantitative effects of this widespread form of damage, especially in natural stone that typifies culturally important sites, but initial results suggest these impacts increase deterioration of sites in the long term [2–4].

Bullet impacts cause compaction and grain fracture directly below the impact, increase surface permeability and reduce surface hardness around the impact [4,5]. Campbell *et al*. [6] showed that impact-induced fractures create 4–7 times more new surface area than the crater itself. Microstructural analysis from below the impact showed open aperture fracture networks decreasing in intensity with distance from the crater floor [6]. These factors aid the ingress of weathering agents such as moisture and salts into the stone, enlarging the region at risk of deterioration [7]. The expansive crystallization of salt from solution widens fracture apertures and pushes grains apart, reducing overall stone strength [8]. Larger fracture apertures and greater porosity also enhance the flow of moisture through capillary rise and surface evaporation. This moisture can lead to dissolution of constituent minerals and cement, further exacerbating stone deterioration through a negative feedback cycle of increasing porosity and decreasing strength [9–11]. It is therefore important for any conservation efforts that the spatial distribution and morphology of surface damage is understood, in order to appreciate the full likely scope of damage, including the sub-surface effects.

Experiments have shown that impact variables such as projectile mass, velocity and angle of impact affect the distribution and morphology of damage. Impact experiments and simulations with projectile trajectories perpendicular to the target face can be constrained with well-defined power-law relationships between impact variables, which are useful in investigating the effects of different factors on resultant damage [12–14]. Perpendicular impacts are generally modelled by spherical stress waves, leading to a symmetric distribution of damage. However, few previous studies have considered the heterogeneity of natural target materials, such as stone [15]. Perpendicular impacts also do not represent a ‘natural’ conflict zone comprehensively, where the angle of impact is likely to be oblique (less than 90°).

The angle of impact affects the morphology of impact damage. Crater shape in hypervelocity impact experiments into loose quartz sand remains circular until impact angles fall below 30°, and in granite targets only elongates at impact angles less than 15° [16]. However, numerical modelling by Pierazzo *et al*. [17] indicates that the locations of peak pressures in the target move down-range and closer to the target surface in oblique impacts, even at incident angles as steep as 60°. This suggests that any degree of obliquity in projectile impact could cause an asymmetric distribution of stress and resultant damage. There is an uncertainty about the applicability of this effect to bullet impacts whose velocities are less than 1000 ms^−1^ and therefore may not produce a comparable shockwave. In hypervelocity experiments, the regions subjected to the strongest asymmetrical stress are typically ejected. The absence of this shockwave in bullet impacts makes asymmetrical craters more likely. Distinguishing oblique from perpendicular impacts on damaged heritage is important for identifying regions that may be more prone to future deterioration.

The aim of this study is to provide quantitative assessment of damage caused by modern rifle bullets in scenarios typical of modern conflict, particularly in the context of sites of cultural heritage. Due to protections surrounding heritage, methods of study need to minimize additional damage or deterioration. Digital photogrammetry provides a means of capturing data from field sites for further analysis, and is a completely non-destructive approach. Such digital means have been useful in recording heritage and mapping decay [18,19]. This study uses digital three-dimensional models of experimental bullet damage into targets consisting of two different types of natural stone, with two different but commonly used types of ammunition. The study compares crater morphology from perpendicular and 45° impacts, and suggests several criteria for differentiating them.

## 2. Material and methods

### 2.1. Target materials and projectile properties

**Figure 1.**
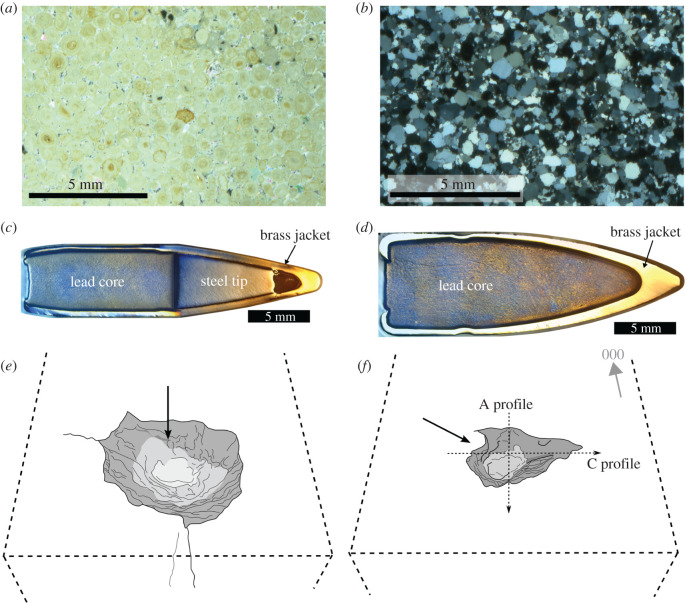
(*a*) Thin section photomicrograph of undamaged Cotswold Hill Cream Limestone under cross polarized light. Spherical ooids can be seen tightly packed, with most pore space infilled with sparry calcite crystals. (*b*) Thin section photomicrograph of undamaged Stoneraise Red Sandstone under cross polarized light. Grains are predominantly quartz, with many exhibiting orange-brown Fe-oxidation rims that have been subsequently overgrown. (*c*) Reflected light micrograph of a 5.56 × 45 mm NATO projectile in cross-section, comprising three parts: a surrounding brass jacket, a steel tip in the nose, and a lead core in the body of the projectile. This differs from the 7.62 × 29 mm (AK-47) projectile (*d*) which has only a brass jacket and fully lead core. (*d*) Reflected light photo micrograph of 7.62 × 39 mm projectile in cross-section (from Campbell *et al*., [6]). (*e,f*) Schematic diagrams (not to scale) showing the angle of impact for perpendicular (90°) and oblique (45°) impacts respectively with relation to the target face. Dashed arrows show the orientation of the A and C profiles measured for each sample.

Freshly quarried cubes (15 × 15 × 15 cm) of Stoneraise Red Sandstone (SRS)(18No.) and Cotswold Hill Cream Limestone (CHCL)(12No.) were selected as target stones because of their analogous lithology and mechanical properties to heritage stones in the Middle East, such as the Mokattam Limestone of Egypt, and the Umm Ishrin sandstones of Petra, Jordan [20–22]. The Cotswold Hill Cream Limestone is an oolitic grainstone from the Middle Jurassic Inferior Oolite (quarried near Ford, UK). The average grain size is 0.5 mm and the porosity approximately 20%. (figure 1*a*). The Stoneraise Red Sandstone is a fine-medium (0.125–0.5 mm), quartz rich sandstone from the Permian New Red Sandstones (quarried near Penrith, UK) (figure 1*b*). It has a porosity of approximately 11% and is generally massive, though some blocks exhibit visible beds of coarser grains (approx. 1 mm).

Controlled firearm experiments were carried out at Cranfield Ordnance Test and Evaluation Centre (Gore Cross, UK) to simulate conflict damage to stone. Two different types of ammunition used in contemporary and past conflicts were fired at incident angles of 90° and 45° to the target face. Firstly, 5.56 × 45 mm NATO (abbreviated in this study as NATO) is a standardized cartridge used in the British SA80 assault rifle, the American M16 family of assault rifles, as well as many other military issue firearms around the world. The second ammunition type is a 7.62 × 39 mm cartridge (abbreviated in this study as AK-47), commonly fired from AK-variant rifles, such as the widely known AK-47. Both ammunition types are a spitzer ogive-nosed projectile with a brass jacket and lead core. The NATO projectile also has a steel tip within the brass jacket (figure 1*c,d*). The AK-47 projectile has a mass of 7.95 grams (123 grains) and the NATO projectile has a mass of 4.04 grams (63 grains). Both cartridges were remotely fired from mounted proof barrels 14 m from the target. Propellant loads for each cartridge were adjusted to reduce velocity and simulate impacts at distances of 200 m (approx. 540 ms^−1^ and approx. 700 ms^−1^ for NATO projectiles). Average engagement distances in urban firefights during the Iraq War ranged from 26 m to over 126 m between combatants, and most soldiers are trained for engagement distances of 0–600 m, so 200 m represents a reasonable distance for simulating impacts in both urban and open scenarios [23,24]. Additional adjustments were made to the AK-47 and NATO cartridges to simulate a range of 400 m (approx. 430 ms^−1^ and approx. 600 ms^−1^, respectively), as well as one shot conducted at standard propellant load (impact at muzzle velocities of approx. 730 ms^−1^ and approx. 930 ms^−1^, respectively). The kinetic energy of the projectile at impact will be in the range 734–2118 J for the AK-47 projectile and 727–1747 J for the NATO projectile. Concrete blocks were placed on all faces, except the target face, for confinement. Target blocks with bedding were oriented so that foliations were parallel to the target face.

A 14-megapixel Fujifilm FinePix S3400 digital camera was used to photograph damaged samples through a 360° rotation at three overlapping camera positions. Samples were then overturned and the process was repeated. The camera was fixed on a tripod approximately 1 m from the sample and its optical zoom (equivalent focal lengths of 24–672 mm) used to ensure sufficient detail was captured. Additional images were taken of the impact crater to ensure adequate capture of morphology. Meshroom (v. 2020.1.1), a free and open-source structure from motion (SfM) pipeline developed by AliceVision, was used to process the approximately 300–400 images for each block into a three-dimensional mesh [25,26]. This number of images was used to ensure the SfM software had enough common reference points and overlap of the entire target block to produce an accurate three-dimensional mesh and texture file because additional observations will be made on fractures in faces other than the target face. In CloudCompare (v. 2.11.3, 2020 [27]), impact damage was isolated from the full block mesh to reduce processing times, then scaled and oriented with the target surface horizontal and an azimuth direction of 000° directed towards the top edge of the block (figure 1*f*).

Depth maps were generated in CloudCompare and further processed in Python. Eighteen cross-section profiles, centred on the deepest point of the crater, were measured at 10° increments. An average profile was calculated for regions with data points from all 18 profiles. Four analogue cross-section profiles, centred on the visually determined deepest point, were measured at 45° increments by placing a 150 mm Barton profile comb across impact craters. The comb profile was then photographed and digitized in QGIS (v. 3.16.0), to be compared to digital profiles along the same orientation to ground truth the models. Once aligned, the Root Mean Square (RMS) difference between the comb profile (*P*_c_) and digital profile (*P*_d_) was calculated using equation (2.1):

2.1 $$\mathrm{RMS}\;\mathrm{difference}=\;\sqrt{\frac{\sum_{i=1}^{n}{\left(P_{c\;i}-P_{d\;i}\right)}^{2}}{n}}.$$

The RMS difference for each sample was normalized (RMS*_N_*) to the maximum profile depth, as measured by the comb profile, to enable comparison between samples:

2.2 $${\mathrm{RMS}}_{N}=\frac{\mathrm{RMS}\;\mathrm{difference}}{\mathrm{Max}\;\mathrm{depth}}.$$

Impact craters were outlined in QGIS from plan view photographs. The edge of the crater was defined visually as the transition point from a depression to undamaged target face. These outlines were analysed in ImageJ (v. 1.53 h) to calculate the crater area, aspect ratio and geometric centre. An area (A) equivalent diameter (*D*_eq_) of the crater area was calculated using:

2.3 $$D_{\mathrm{eq}}=2\sqrt{\frac{A}{\pi }}.$$

## 3. Results

All samples experienced the loss of material and the formation of an impact crater which contained fine grained, powdery material and a pale discoloration in the central region. Crater size and morphology differ between lithology, angle of impact and projectile type.

### 3.1. 90° Impact trajectory

**Figure 2.**
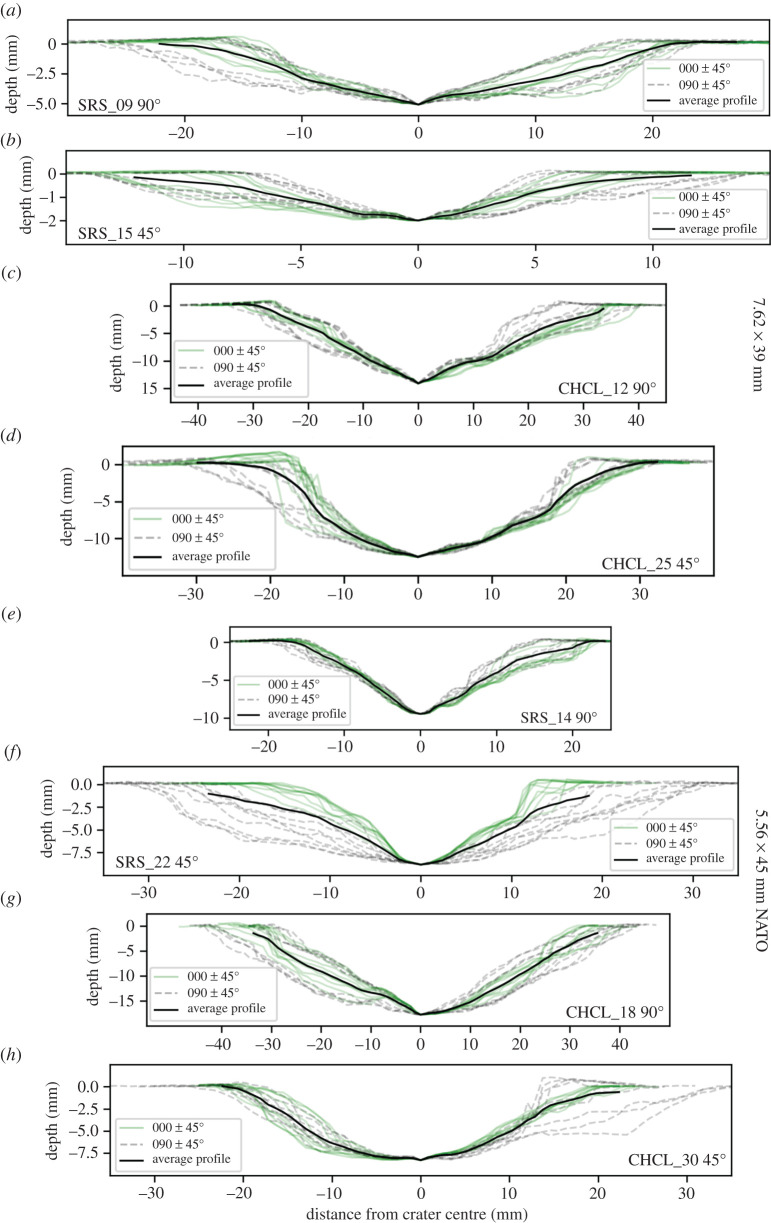
Summary of 18 cross-sections for each sample (*a*–*h*). Profiles ±45° to 000° are coloured green, while profiles ±45° to 090° are dashed grey. Note the asymmetry imposed by the 45° impact in sample SRS_22 (*f*). Profiles with similar azimuth to projectile trajectory (grey dash) have a wider diameter and shallower slopes, while profiles orthogonal to this (green) have steeper slopes and a narrow diameter. Other angled impacts show up-range sides dipping more steeply than the down-range ones (e.g. sample CHCL_25 (*d*) and CHCL_30 (*h*). Incipient spall fragments can be identified as areas raised above 0 mm depth on crater edges (e.g. between −20 and −10 mm on sample CHCL_25 (*d*) and +15 mm on sample CHCL_30 (*h*). Direction of projectile is left to right for 45° impacts (*b,d,f,h*). Profiles and depth maps of all samples can be found in supplementary data.

Sandstone targets shot with AK-47 projectiles have shallow, bowl-shaped craters with an average depth of 4.62 mm and an average diameter of 33.76 mm (table 1). An average aspect ratio of 1.10 supports visual observations of a roughly circular shape. There are few visible surface fractures surrounding the craters, but if present, they are short and appear closed. Within and around some craters there is a dark grey discoloration from the lead within the projectile. The cross-section profiles through impacts have a rotational symmetry around the centre of the crater (figure 2).

**Table 1.** Summary of the average crater parameters for each firing condition and description of visible surface fractures on the target. *d* = max depth, *D*_eq_ = area equivalent diameter.

ammunition/projectile	target	angle of impact (°)	*d* (mm)	*D_eq_* (mm)	aspect ratio	fractures
5.56 × 45 mm NATO	limestone	90	15.65	63.55	1.18	radial, open aperture fractures to the edge of the block
		45	10.28	46.32	1.20	open aperture fractures mostly extend to edge of block
	sandstone	90	13.29	50.82	1.17	radial fractures with open apertures in some samples
		45	6.48	35.53	1.45	few very short fractures with closed apertures
7.62 × 39 mm (AK-47)	limestone	90	23.95	73.97	1.08	open radial fractures to the edge of the block. spall fragments bordered by narrow fractures concentric with crater edge
		45	8.99	41.22	1.21	one sample has open aperture fractures to the edge of the block
	sandstone	90	4.62	33.76	1.10	short fractures with closed apertures
		45	3.13	21.42	1.16	narrow aperture fractures to the edge of the block in some samples

**Figure 3.**
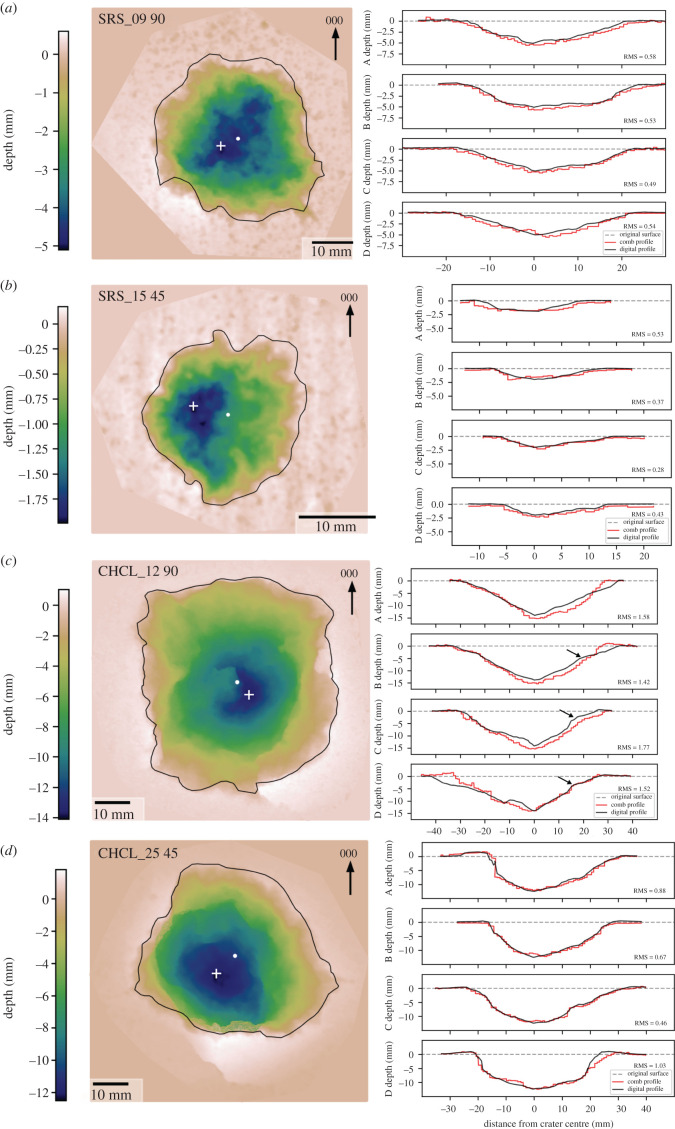
Depth maps (left) of impact craters caused by 7.62 × 39 mm (AK-47) projectiles into blocks of Stoneraise Red Sandstone (SRS) (*a,b*) and Cotswold Hill Cream Limestone (CHCL) (*c,d*). Black line is the crater outline, the white cross (+) is the deepest point of the crater, and the white circle marks the geometric centre. Black arrows indicate the change of slope between central depression and outer spall zone. Direction of projectile is left to right for 45° impacts. Adjacent to each depth map (right) is a comparison of digital (black line) and analogue comb (red) profiles taken at 45° intervals starting at 000° (labelled A–D). The dashed grey line shows the original undamaged target face. Root mean square difference (RMS) values are in mm. Profiles and depth maps of all samples can be found in supplementary data.

Limestone targets shot with the same projectile type have deeper (23.95 mm) and wider (73.97 mm) impact craters than sandstone targets. The crater morphology is composed of two regions, a steep-sided central region, surrounded by a shallower dipping spall region, separated by a change in slope (arrows in figure 3*c*). Some impacts have prominent radial fractures emanating from the crater edges, in some instances with apertures several mm wide and extending to adjacent faces. Other samples only have one or two radial fractures with narrow apertures (approx. 1 mm), which can also extend to the edge of target face. Some samples have incipient spall fragments that are raised above the target face. They are bordered by very narrow aperture fractures that are roughly concentric to the crater edge.

Impacts into sandstone targets with NATO projectiles produced craters on average 2.9 times deeper (13.29 mm versus 4.65 mm) and 1.5 times wider (50.82 mm versus 33.76 mm) than AK-47 projectile impacts. Craters have a slightly higher aspect ratio of 1.17, and a crater outline that tends towards a square shape. Some craters have radial fractures with visible open apertures away from the crater. The steel tip from the projectile remains embedded in the crater floor. Crater profile morphology is more complex than the simple bowl craters observed in AK-47 projectile impacts. Profiles have a central region with steep sides and shallow dipping outer spall region, similar to impacts of AK-47 projectiles into limestone targets.

Limestone targets shot with NATO projectiles have, on average, shallower (15.65 mm) and narrower (63.55 mm) craters than those shot with AK-47 projectiles. All craters have open aperture radial fractures that extend to the edge of the block, though apertures are not as wide as seen in AK-47 projectile impacts. There are metal smears and grey lead deposits at the base of the craters, with the steel tip either embedded or absent, leaving a small central depression at the base of the crater. This depression is reflected in the cross-sectional profiles as a vertical sided pit at the middle of the profile (see supplementary data). Crater morphology is similar to other impacts in that it has a steep sided central region surrounded by a shallower dipping spall zone.

### 3.2. 45° impact trajectory

Sandstone targets impacted with AK-47 projectiles at 45° have extremely shallow (3.13 mm) craters with an average diameter of 21.42 mm. Crater shape is still roughly circular with an aspect ratio of 1.16, though this is slightly larger than perpendicular impacts at the same conditions. Cross-sections along the same axis as the projectile trajectory (C Profiles) show an asymmetry in morphology. They have a shorter, steeper wall on the up-range (toward 270°) side and a longer shallower wall on the down-trajectory side (toward 090°). The morphology of the orthogonal A profile is more symmetrical. Dark-grey lead residue is present on down-range regions of the crater edge and adjacent to the crater on the target face.

**Figure 4.**
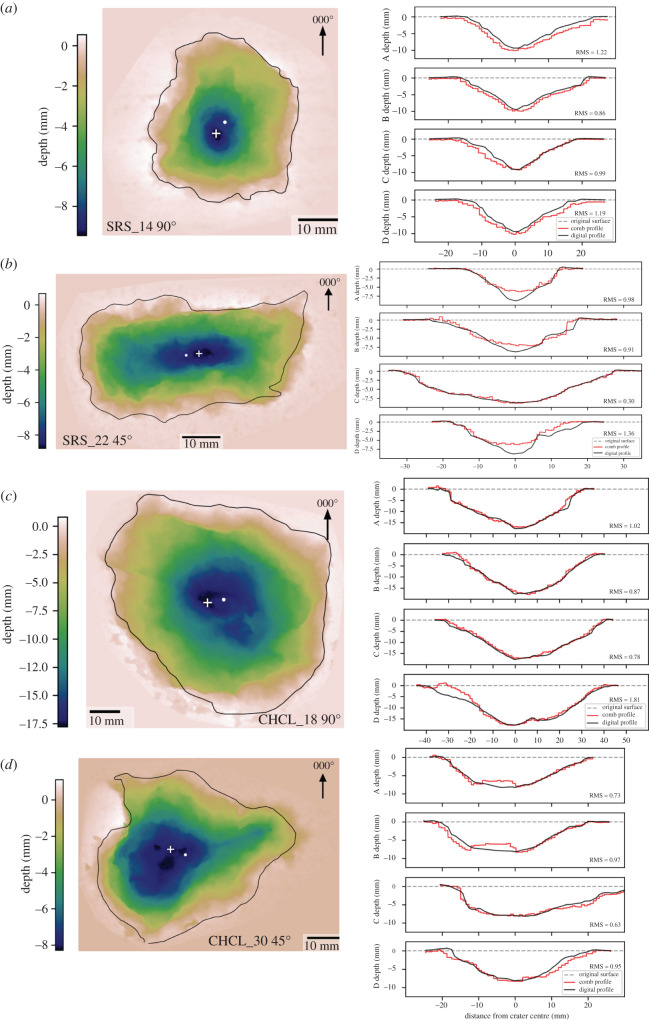
Depth maps (left) of impact craters caused by 5.56 × 45 mm NATO projectiles into Stoneraise Red Sandstone (*a,b*) and Cotswold Hill Cream Limestone (*c,d*). Black line is the crater outline, the white cross (+) is the deepest point of the crater, and the white circle marks the geometric centre. Direction of projectile is left to right for 45 °impacts. Adjacent to each depth map (right) is a comparison of digital (black line) and analogue comb (red) profiles taken at 45° intervals starting at 000° (labelled A–D). The dashed grey line shows the original undamaged target face. Root mean square difference (RMS) values are in mm. Profiles and depth maps of all samples can be found in supplementary data.

Limestone targets impacted by AK-47 projectiles at 45° have the highest aspect ratio (1.24) of all samples across both rock types impacted using this projectile. The impact craters are on average shallower (8.99 mm) and smaller in diameter (41.22 mm) than perpendicular impacts into the same target material. The two samples shot under these conditions are quite different. One sample shot at 45° (CHCL_25) has incipient spall fragments at the crater edge, and open aperture fractures that extend from the crater to the edge of the block. The other sample (CHCL_28) shot under the same conditions has no radial fractures around the crater and a greater difference between analogue and digital profiles (table 2 and figure 4).

**Table 2.** Average of normalized root mean square difference (RMS_N_) between profiles obtained via the Barton comb method and from digital photogrammetry models.

ammunition/projectile	target	average RMS_N_ difference (%)
NATO	limestone	6.0
		10.1
	sandstone	9.2
		9.7
AK-47	limestone	13.1
		15.7
	sandstone	17.8
		14.2

Oblique impacts of NATO projectiles into sandstone targets resulted in the most elongate craters of any conditions, with an average aspect ratio of 1.45. Crater depth averages 6.48 mm and average diameters 35.53 mm. These craters are larger than in sandstone targets impacted by AK-47 projectiles at both angles of impact. The long axis of the crater is (sub) parallel to the trajectory of the projectile. The cross-section profiles reflect the higher aspect ratio, with profiles ± 45° of the A profile (000°) showing a narrower, steeper sided cross-section, while profiles ± 45° of the C profile are wider with shallower dipping sides (figure 2*f*).

Much like other angled impacts, NATO projectiles produce shallower (10.28 mm) and narrower (46.32 mm) impact craters in limestone targets than perpendicular impacts with the same projectile. Impact craters under these conditions generally fall into two groups based on crater morphology. The first group has many, open aperture fractures radiating from the crater to the edge of the target block. Fracture orientations are predominantly between 000° and 180°, i.e. in the down-range half of the block. This group also has multiple incipient spall fragments bordered by fractures concentric to the crater. The second group have fewer or no radial fractures visible, the occasional incipient spall fragment, and most have metal smears or traces of the projectile on the crater floor. Both groups show a distinct asymmetry in the C profile, with a short, steep up-range wall and a longer shallow dipping down-range one.

## 4. Discussion

There is a stark contrast in the crater morphology caused by AK-47 projectile impacts into sandstone and limestone targets. For perpendicular impacts, sandstone targets have simple, shallow, bowl-shaped craters, whereas limestone targets exhibit a two-part structure of steep sided central pit and shallow dipping outer spall region (dish shaped). This two-part crater morphology is similarly observed during hypervelocity experiments into multiple target lithologies [28–30]. Reported strength values for similar rock types (Mokattam Limestone versus Umm Ishrin Sandstone) suggest the limestone targets have lower compressive (40 MPa versus 105 MPa) and tensile strengths (1.3 MPa versus 2.84 MPa) than the sandstone targets [21,31,32]. The restriction of spallation to the limestone targets, despite their higher porosity (and thus stress wave attenuation), is consistent with their lower tensile strength. Limestone targets have longer radial fractures with wider apertures than observed in sandstone targets, possibly linked to their greater porosity (20% versus 11%) [33].

All limestone targets had deeper and wider crater dimensions than in sandstone targets shot with the same conditions (table 3). The different response of the two lithologies is due to the target properties, but pinpointing the exact variable is difficult from the experiments presented here. There is a paradoxical relationship in hypervelocity experiments between porosity, strength, and crater size. Increased porosity, when viewed independently of stone strength, acts to decrease crater size through the dissipation of energy during pore space collapse [34]. Compressive strength of the target lithology has a similar effect on crater size: the stronger the target material, the smaller the impact craters. However, increasing porosity decreases compressive strength, so while the decreased strength acts to increase crater size, the increased porosity acts to counter this [34,35]. The lower velocities (∼400–900 ms^−1^) of the experiments presented here may preclude a shockwave forming upon impact. In this case, momentum transfer from the projectile to the target controls crater excavation. Momentum transfer is influenced by both target and projectile properties. The steel tip of the NATO projectile is embedded, relatively intact, in the crater floor of some targets, because it is stronger than the all lead core of the AK-47 projectiles. The impact energy of both projectiles is similar, yet NATO projectiles result in larger crater volumes, suggesting projectile properties have caused greater momentum transfer to the target. The considerable differences between the damage caused in the two different rock types emphasizes that consideration of target material properties, particularly the tensile strength, is a key aspect of evaluating and understanding bullet impact damage.

**Table 3.** Ratios of the limestone (CHCL) to sandstone (SRS) crater dimensions show that for all conditions, limestone targets exhibit deeper and wider craters to sandstone counterparts. *d* = max depth, *D* = area equivalent diameter.

angle of impact	AK-47		NATO	
	*d_CHCL_/d_SRS_*	*D_CHCL_/D_SRS_*	*d_CHCL_/d_SRS_*	*D_CHCL_/D_SRS_*
90°	5.19	2.19	1.18	1.25
45°	2.87	1.92	1.59	1.30

Oblique impacts can be distinguished from perpendicular impacts where stone type and projectile are the same. Perpendicular impacts are deeper and wider than comparable angled impacts, a pattern also observed in hypervelocity experiments with increasing obliquity [30]. This is due to perpendicular impacts transferring more kinetic energy to the target than oblique trajectories. Projectiles with oblique trajectories are more likely to ricochet, retaining kinetic energy that would be transferred to the target in a perpendicular impact [16]. Crater size is also linked to the kinetic energy of the projectile, so with less energy transfer, the maximum stress values experienced in the target may not exceed its strength, resulting in less fracturing and smaller crater dimensions [36–38].

All the perpendicular impact craters have a broadly symmetrical distribution of damage around the point of impact, with no clear asymmetry in crater profiles. The planform crater shape is roughly circular, but spallation of plate-like clasts from crater edges has modified the crater outlines so that the aspect ratios diverge from 1 (perfectly circular) by average values up to 0.18. This modification has been observed in other impact experiments into natural stone [29]. 45° impacts have higher aspect ratios than perpendicular impacts under similar conditions, but in many cases this difference is small, e.g. NATO projectiles into limestone targets (1.20 versus 1.18) (table 1). A threshold of aspect ratios for characterizing obliquity can be defined for given targets and projectiles (table 1). NATO projectile impacts into sandstone targets have the highest aspect ratios, evident from the narrower diameters of the craters in A profiles compared to the C profiles (figure 2).

In hypervelocity experiments involving granite targets, crater elongation does not occur until impact angles fall below 15°, and for loose sediment targets, less than 30° [16]. This is due to hypervelocity impacts producing a hemispherical shock wave which drives the excavation of material from the crater [39]. The symmetry of the shockwave results in circular impact craters except for very low incidence angles. The velocity range (approx. 400–900 ms^−1^) of the experiments presented here may be too slow to generate a hemispherical shock wave, so circularity may not occur in moderately inclined impact trajectories. Crater elongation is observed here for some conditions at impact angles of 45°, and cross-section profiles in line with the impact trajectory (C profiles) show the same steep up range slope and shallow down range slope as very oblique hypervelocity experiments [16,40]. Wallis *et al*. [40] show that subtle asymmetry is present in impacts with even a small degree of obliquity, though these impacts were into aluminium plates, which may not be directly comparable because of their ductile deformation. The Wallis *et al.* [40] study does indicate that any obliquity can cause an asymmetric distribution of damage, suggesting that some regions of stone surrounding the impact will be at risk of faster deterioration than others.

To properly combat future deterioration of impacted stone from weathering, it is important to understand the type of damage and its location. These experiments show that asymmetric cross-section profiles, shallower and smaller craters, and higher aspect ratios characterize oblique projectile trajectories. Analogue measurement of profiles using a Barton comb is a simple and cheap way of collecting data in the field. This method can provide measurements of crater depth, diameter and morphology, without additional processing. The method is however limited to sites and impact damage that are accessible to the researcher. Photogrammetry as an alternative is more versatile in terms of site accessibility and safety: aerial drones can successfully obtain imagery without direct access to a site [18]. Photogrammetry also preserves a digital record of the damage, that can be used to monitor change over time or measure additional variables [6,41,42]. On the other hand, this method requires more post-collection processing to turn photographs into usable three-dimensional models, which can be time-consuming depending on the number of images and computing power available [43]. This study used between 300 and 400 images to generate three-dimensional models of the target blocks in their entirety; this number could be reduced and still produce usable models. Gilbert *et al*. [5] and Campbell *et al*. [6] created and analysed a good-quality three-dimensional model generated from only 142 images. Even fewer images could be used if only the impact crater was modelled.

Analogue collection methods for crater profiles allows the digital models to be ‘ground-truthed’ to damage observed in the field. The normalized root mean square difference (RMS_N_) between the profiles produced by analogue and photogrammetric methods range from 6.0% to 17.8%. There are several factors to consider when evaluating the difference between analogue and digital profiles. Firstly, the Barton comb has a limited number (approx. 150) of teeth with a set width (approx. 1 mm), creating a stepped profile that can miss subtle changes in the crater morphology. The digital profile method interpolates between the point cloud data, allowing as many sample points along the profile as desired. This results in a much smoother profile, so even without any other source of difference, profiles from the two methods would not match perfectly. The normalized difference for deeper craters (e.g. those impacted with NATO projectiles) is consistently around 10%. Profiles from both methods are in reasonable agreement with each other, and therefore either method is a viable choice, depending on the specific research aims and conditions of the field site.

The use of non-destructive methods for assessing stone is invaluable for fragile and damaged heritage. For oblique impacts, internal damage, such as fracturing, may be more intense in a downrange direction, as suggested by the shift in the location of peak pressures experienced with increasing obliquity [17]. The use of damage morphology to identify asymmetry and infer a possible downrange direction is a useful first approach to identify regions at risk. Following up with further non-destructive methods, such as surface hardness, ultra-pulse velocity, and surface permeability measurements can identify and corroborate damage surrounding impacts [2,4,5]. This would identify vulnerable regions at risk of increased capillary rise and salt-driven deterioration, allowing for more comprehensive and specific weathering risk assessments to be made. Such detailed assessments will prove invaluable to the conservation efforts of culturally important sites, especially those recently affected by armed conflict. In these areas, the opportunity for high-resolution and highly technical investigations may be (temporarily) limited. This method could support relatively fast and inexpensive first-response documentation and interpretation of damage for those working *in situ*.

## 5. Conclusion

In experiments to investigate the surface damage caused by rifle bullets for conditions simulating modern conflicts, impacts excavated craters with diameters from 22 to 74 mm and depths from 3 to 24 mm. In all conditions, limestone targets had larger crater dimensions than sandstone targets. Limestone targets also exhibit a more complex, two-part crater morphology consisting of a central excavation surrounded by a shallow dish shaped spall zone, compared to the simple bowl shape of the sandstone craters. Limestone targets had a higher occurrence of radial fractures extending from the crater to the edge of the block than sandstone targets. Target properties are a major factor in determining the extent and distribution of bullet and shrapnel damage, suggesting building stone with low tensile strengths are at greater risk of significant damage.

Impacts with an incident angle of 45° produced craters that were shallower and narrower than experiments shot at 90°. Oblique impacts also caused asymmetrical crater profiles, with a steep dipping up range slope and a shallower dipping down range one. Differences between perpendicular and oblique impact damage are quantifiable: for example, crater aspect ratios can distinguish perpendicular from oblique impacts for given target and projectile types. Of the two ammunitions used, the 5.56 × 45 mm NATO projectiles produced larger and deeper craters than the 7.62 × 39 mm (AK-47) projectiles, as well as causing the most prominent asymmetry in crater profiles and outlines for oblique impacts.

This characterization of damage common to contemporary conflicts, with a focus on cultural heritage caught in the crossfire, is important for the conservation of affected sites.

## Data Availability

The datasets supporting this article have been uploaded as part of the electronic supplementary material [44].
